# Inactivating mutations in genes encoding for components of the BAF/PBAF complex and immune-checkpoint inhibitor outcome

**DOI:** 10.1186/s40364-020-00206-3

**Published:** 2020-07-16

**Authors:** Kevin Courtet, Yec’han Laizet, Carlo Lucchesi, Alban Bessede, Antoine Italiano

**Affiliations:** 1 INSERM U1218, Bordeaux, France; 2 Bioinformatic Unit, Institut Bergonié, Bordeaux, France; 3 Immusmol, Bordeaux, France; 4 University of Bordeaux, Bordeaux, France; 5 Early Phase Trials and Sarcoma Units, Institut Bergonié, 229 cours de l’Argonne, 33076 Bordeaux Cedex, France

**Keywords:** Immune checkpoint inhibitors, SWI/SNF complex, BAP/PBAF complex

## Abstract

Alterations of genes encoding subunits of the BAF/PBAF complexes are among the most frequent gene aberrations in human cancer. Such alterations have been shown to have an impact on tumor microenvironnement and on the capacity of tumors to respond to immune-checkpoint inhibitors (ICI). We analysed the clinical and genetic data from 43,728 patients accessed through cBioportal. The mutational frequencies of ARID1A, ARID1B, ARID2, PBRM1, SMARCA4, and SMARCB1 were 6.6%, 3,4, 3.4, 3.2, 4.1, and 1.2%, respectively. We then investigated the association between the presence of least one nonsynonymous somatic mutation of ARID1A, ARID1B, ARID2, PBRM1, SMARCA4, or SMARCB1 and overall survival of 1661 patients treated with an ICI. Across the entire cohort, patients with BAF/PBAF mutated tumors have a statistically significant improvement in overall survival (median overall survival: 28 months [95% CI 21.6–34.3] versus 15 months [95% CI 12.9–17.0], *p* < 0.0001). When tumor mutational burden was adjusted for a multivariable Cox regression analysis, BAF/PBAF gene mutations remained an independent prognostic factor for overall survival in patients treated ICI. Our results establish a relationship between mutations in key genes encoding for components of the BAF/PBAF complex and outcome of patients treated with ICI. Further studies are needed to elucidate the underlying mechanisms of this interaction.

**To the Editor**

Two recent pre-clinical studies have shown that alterations of genes encoding subunits of polymorphic BRG−/BRM-associated factor (BAF) and Polybromo-associated BAF (PBAF) complexes may have an impact on tumor microenvironment and on the capacity of tumors to respond to immune-checkpoint inhibitors (ICI) [1, 2].

Patients were selected from the cBioPortal for Cancer Genomics (http://cbioportal.org) which provides a digitalized resource for investigating cancer genomics data and their correlation with clinical outcome [3, 4]. At the time of analysis, genetic data were available for 43,728 patients whereas survival and genetic data were available for 29,531 cancer patients of whom 1661 were treated with an ICI regimen [4]. Overall survival (OS) of ICI patients was defined as the time from the first infusion of treatment until death or last patient contact. Survival rates were estimated using the Kaplan–Meier method. Prognostic factors were planned to be identified by univariate and multivariate analyses using a Cox regression model. Variables tested in univariate analysis included: age, gender, tumor type, tumor mutational burden, and presence of *BAF/PBAF* gene mutation. Analyses were performed using SPSS 25.0 statistical software (IPSS Inc., Chicago, USA). All statistical tests were two-sided, and *p* < 0.05 indicated statistical significance.

We first investigated the prevalence of nonsynonymous somatic mutation of the *BAF/PBAF* genes: *ARID1A*, *ARID1B*, *ARID2*, *PBRM1*, *SMARCA4*, and *SMARCB1* in 43,728 patients with different cancer types (Fig. 1a). Patients with uterus carcinoma, melanoma, and bladder cancer have the highest proportion of *BAF/PBAF* gene mutations.

**Fig. 1:**
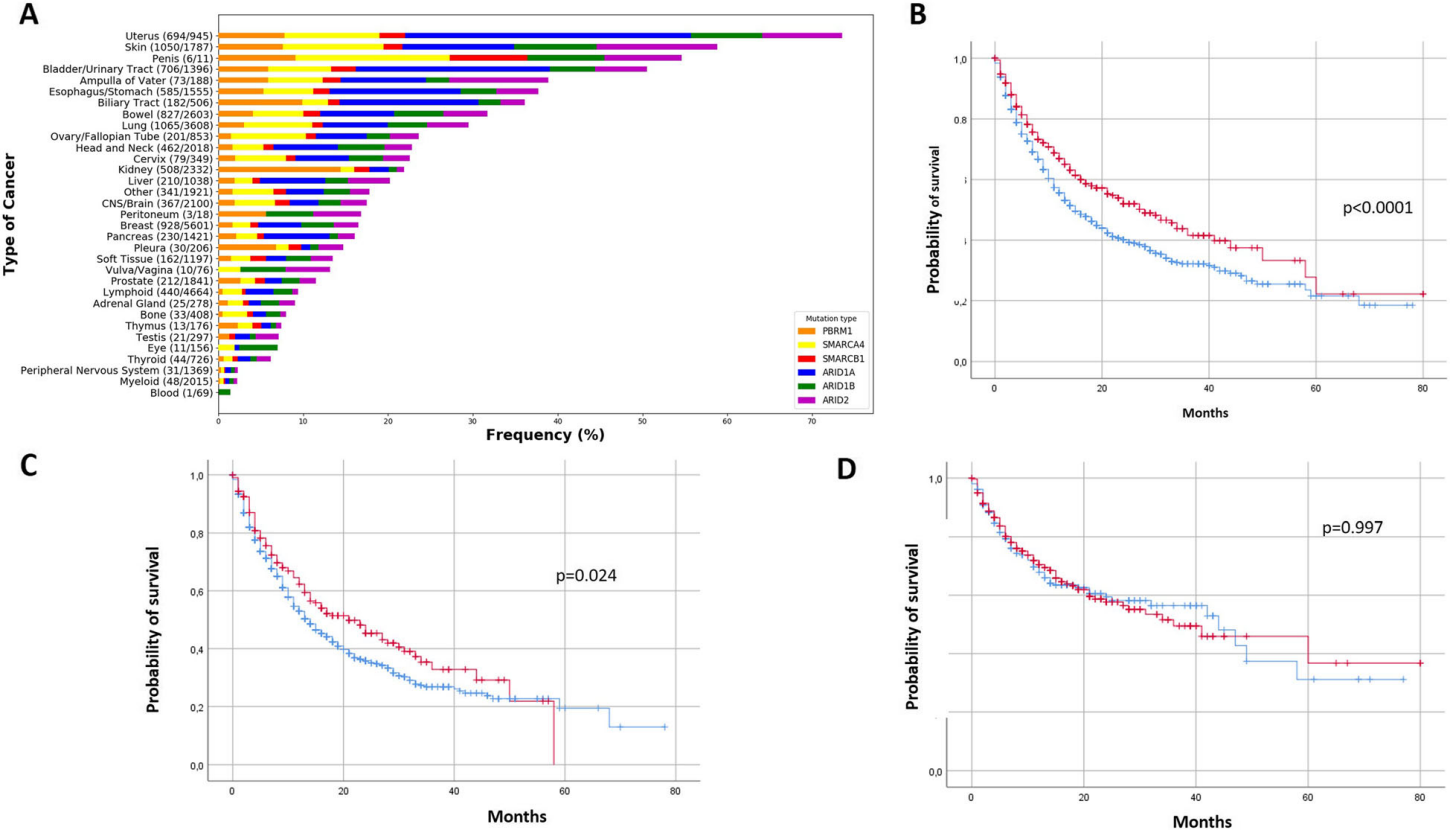
Prevalence of *BAF/PBAF* genes mutations in 43,728 patients with different cancer types (**a**) and overall survival of patients treated with immune-checkpoint inhibitors (ICI) according to BAF/PBAF mutational status and tumor mutational burden (**b**-**d**). **a** Prevalence of *ARID1A*, *ARID1B*, *ARID2*, *PBRM1*, *SMARCA4*, or *SMARCB1* genes mutations in 43,728 patients with different cancer types (**b**) Kaplan-Meir curves of overall survival in 1661 patients treated with (ICI) according to *BAF/PBAF* gene status for the overall cohort (**b**) and in patients with low (< 10 mutations/megabase) (**c**), and high (> 10 mutations/megabase) (**d**) tumor mutational burden. Red curves: BAF/PBAF mutated tumors, blue curves: *BAF/PBAF* wild-type tumors

We then investigated the association between the presence of least one nonsynonymous somatic mutation of these genes and overall survival in a previously described cohort of 1661 patients treated with a ICI. We found that patients with BAF/PBAF mutated tumors have a statistically significant improvement in overall survival (OS) (median OS: 28 months [95% CI 21.6–34.3] versus 15 months [95% CI 12.9–17.0], *p* < 0.0001) (Fig. 1b).

To investigate the possibility that this result might simply be attributable to an intrinsic prognostic impact of BAF/PBAF mutations, unrelated to ICI, we analysed the outcome of 27,870 patients with metastatic cancers who did not receive ICI and with available survival data. In these patients, BAF/PBAF mutated tumors had worse OS in comparison with wild-type tumors (109.2 months versus 61.8 months, *p* < 0.001) suggesting that the improved outcome observed in patients treated with ICI was related to a real predictive value of BAF/PBAF aberrations (Supplementary Figure 1).

When analysing the prognostic impact of BAF/PBAF mutations according to tumor mutational burden (TMB) level, *BAF/PBAF* mutations were associated with overall survival only in tumors with low TMB (< 10 mutations/megabase) (21 months versus 14 months, *p* = 0.024) (Fig. 1c-d) who represented the majority of patients (*n* = 1173, 70.6%). When TMB was adjusted for a multivariable Cox regression analysis in the whole cohort, *BAF/PBAF* gene mutations remained an independent prognostic factor for OS in patients treated ICI (Table 1).

**Table 1:** Multivariate analysis for overall survival (*n* = 1661)

		HR	95% CI	*P* value
**Tumor mutational burden**	≤ 10 mutation/Mb	1.47	[1.2–1.8]	< 0.0001
	> 10 mutation/mb	1		
**Cancer Type**	Bladder	1.04	[0.8–1.3]	< 0.0001
	Melanoma	0.55	[0.4–0.7]	
	NSCLC	1.3	[1.1–1.5]	
	Other	1		
**BAF/PBAF mutational status**	Wild-type	1.2	[1.05–1.4]	0.017
	Mutated	1		

The presence of *BAF/PBAF* gene mutations was associated with improved outcome on immunotherapy in almost all carcinomas types except in non-small cell lung cancer (NSCLC), unknown primary carcinoma and renal cancer even if the effect for some cancer types did not reach statistical significance, due to limited sample size. Interestingly, a recent study has shown that mutation or low expression of *BAF/PBAF* genes including *SMARCA2* and *PBRM1* were associated with higher neoantigen burden and higher tumor infiltration of activated CD8 T-cells in NSCLC [4]. These data indicate that the impact of *BAF/PBAF* gene aberration on tumor microenvironment may vary according to tumor types and further studies are needed to elucidate the underlying mechanisms of this interaction.

Altogether, our findings establish a relationship between mutations in key genes encoding for components of the BAF/PBAF complex and outcome of patients treated with ICI and pave the way for clinical trials combining ICI with small-molecule inhibitors of chromatin remodeling pathways such as EZH2 inhibitors [5].

## Supplementary information

**Additional file 1: Supplementary Figure 1.** Kaplan-Meier curves of overall survival of 27,870 cancer patients according to BAF/PBAF mutational status (red curves: mutated BAF/BPAF, blue curves: wild-type BAF/PBAF).

## Data Availability

Not applicable.

## Abbreviations

BAF: Polymorphic BRG−/BRM-associated factor
ICI: Immune checkpoint inhibitors
NSCLC: Non-small cell lung cancer
OS: Overall survival
PBAF: Polybromo-associated BAF (PBAF)
TMB: Tumor mutational burden
