# Supplementary material for: Inactivating mutations in genes encoding for components of the BAF/PBAF complex and immune-checkpoint inhibitor outcome
Source: Biomark Res. 2020 Jul 16;8:26. doi: 10.1186/s40364-020-00206-3 (PMC7366303; doi:10.1186/s40364-020-00206-3)
Supplement: Supplementary file 1 — Additional file 1: Supplementary Figure 1. Kaplan-Meier curves of overall survival of 27,870 cancer patients according to BAF/PBAF mutational status (red curves: mutated BAF/BPAF, blue curves: wild-type BAF/PBAF). [file 40364_2020_206_MOESM1_ESM.docx]

**Supplementary Figure**


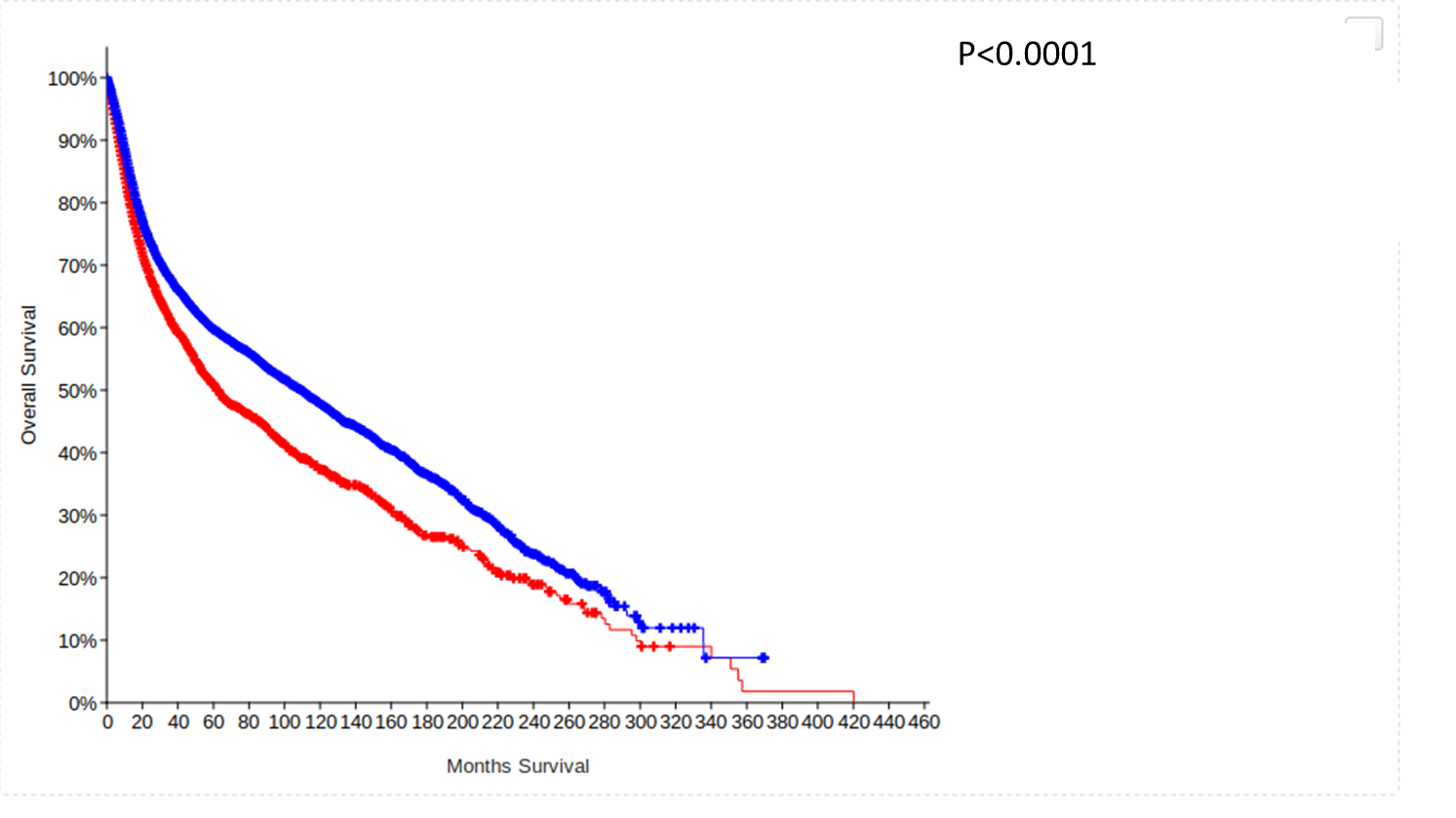


**Supplementary Figure 1**. Kaplan-Meier curves of overall survival of 27870 cancer patients according to BAF/PBAF mutational status (red curves: mutated BAF/BPAF, blue curves: wild-type BAF/PBAF)
